# Acute side effects of proton and photon radiotherapy for medulloblastoma: a retrospective national multicenter study

**DOI:** 10.1007/s11060-025-05016-x

**Published:** 2025-04-15

**Authors:** Linn Söderlund Diaz, Måns Agrup, Anna Asklid, Anna Embring, Jacob Engellau, Ingrid Fagerström Kristensen, Anna Flejmer, Charlotta Fröjd, Martin P Nilsson, Anna Maja Svärd, Angelica Walfridsson, Malin Blomstrand

**Affiliations:** 1 Department of Oncology, Sahlgrenska University Hospital, Blå Stråket 2, Gothenburg, 413 46 Sweden; 2 Department of Oncology, Linköping University Hospital, Linköping, Sweden; 3 Department of Oncology, Karolinska University Hospital, Stockholm, Sweden; 4 Department of Oncology-Pathology, Karolinska Institute, Stockholm, Sweden; 5 Department of Radiation Oncology, Skåne University Hospital, Lund, Sweden; 6 Department of Radiation Oncology, Uppsala University Hospital, Uppsala, Sweden; 7 Department of Oncology, Umeå University Hospital, Umeå, Sweden; 8 Sahlgrenska Academy, Institute of Clincial Sciences, Department of Oncology, Gothenburg, Sweden

**Keywords:** Medulloblastoma, Craniospinal irradiation, Proton radiation, Acute toxicity, Hematologic toxicity.

## Abstract

**Purpose:**

A comparison of acute toxicity between photon and proton radiotherapy (RT) for children undergoing treatment for medulloblastoma.

**Methods:**

This retrospective multi-institutional cohort study included 96 children < 18 years treated for medulloblastoma in Sweden during 2008–2020. Patients treated with protons (*n* = 37) and photons (*n* = 59) were compared regarding acute side effects and radiation dose to intracerebral organs at risk (OARs). Data was collected from a prospectively maintained national database and was supplemented from a retrospective review of medical records. Acute symptoms were graded according to CTCAE (Common Terminology Criteria for Adverse Events), maximum grade occurring during RT and within 2 months after RT. Hematological toxicity was evaluated according to maximum grade and percentual reduction during RT.

**Results:**

No significant differences in incidence or severity of acute symptoms were observed between proton-RT and photon-RT; grade ≥ 2 fatigue (5.4 vs. 10.2%), headache (2.7 vs. 3.4%), nausea (43.2 vs. 42.4%), dermatitis (5.4 vs. 15.3%), gastrointestinal toxicity (0 vs. 0%), weight loss (10.8 vs. 8.5%). Median percentual reduction (0% vs. -11,25%) in hemoglobin was significantly smaller during proton-RT (*p* < 0.001). No difference was observed for leucocytes, neutrophiles, or platelets. Absorbed mean dose to intracranial OARs was significantly lower with proton-RT.

**Conclusion:**

This is one of the largest studies comparing acute side effects of proton-RT and photon-RT including only paediatric medulloblastoma patients. Proton-RT was safe and well tolerated regarding acute side effects. Absorbed dose to intracranial OARs was significantly lower with proton-RT. Further investigations of long-term side effects and cognitive evaluation is needed to show that this will translate into true clinical value for patients.

## Introduction

Medulloblastoma is the most common malignant paediatric central nervous system (CNS) tumor [1–5]. With modern post-surgical therapy including craniospinal irradiation (CSI) with boost and chemotherapy, the survival rate is 70–85% [2]. Though the prognosis has improved, medulloblastoma survivors often experience significant treatment related side effects such as neurocognitive decline, hearing impairment, hormonal deficiency, reproductive problems, growth defects and secondary cancers [3, 5–8]. Until recently medulloblastoma was treated with photon radiotherapy (RT), but the use of proton-RT for curable brain tumors such as medulloblastoma has steadily increased worldwide [9, 10]. Proton therapy enables a highly conformal target coverage by using the physical advantage of the Bragg-peak, while sparing normal tissue from radiation dose [2, 10–14].

Dosimetric studies have demonstrated that CSI with protons significantly spare normal tissue anterior to the vertebral body such as heart, lungs, thyroid gland, liver, and kidneys compared with photon RT [2, 15–17]. Proton therapy also enables reduced dose to healthy brain tissue beyond boost margins and spare critical intracranial structures such as the cochlea, pituitary gland, hippocampi and temporal lobes [1, 12, 15].

Despite the increasing use of proton CSI therapy for medulloblastoma there is still limited clinical data on both acute and long-term side effects of the treatment [10]. A few studies in recent years have shown an advantage of proton CSI therapy regarding acute side effects such as lower incidence of nausea [10, 18], weight loss [18] and hematological toxicity [9, 18]. However, since medulloblastoma is a rare disease and the availability of proton treatment has been limited, studies on outcome of proton CSI therapy are often small. The last decade proton treatment has become the new standard of care for medulloblastoma patients in Sweden. This retrospective analysis on prospectively registered data in a national radiotherapy register aims to compare outcome and acute toxicity associated with photon and proton CSI treatment for medulloblastoma in a larger population.

## Materials and methods

### Data source and cohort

Cases were identified from the RADTOX Quality Registry, which is a prospectively maintained Swedish national RT registry for children with almost 100% coverage [19]. The registry contains demographic data (gender, age at diagnose and performance status), physician-graded acute and late side effects and RT dose distribution (maximum and mean dose to target and OARs) [20].

In total, 105 patients < 18 years were treated with RT for medulloblastoma during the years 2008–2020. Following exclusion of 9 patients who received a combination of photon-RT and proton-RT, 96 patients remained and constituted the present study population. Patients were treated with either photons (*n* = 59) or protons (*n* = 37). Until 2015 all patients were treated with photon-RT. From 2016 when proton therapy became accessible in Sweden most patients have received proton-RT.

To complement the data from the RADTOX Quality Registry medical records were retrospectively reviewed and the following information was collected: molecular and genetic subgroup, follow-up MRI (Magnetic Resonance Imaging) and site of relapse, acute side effects, use of nasogastric tube, weight loss, and blood counts during treatment. For analysis of dose distribution to cranial OARs, hippocampus and temporal lobes were retrospectively contoured by the investigator.

Patients were categorized into standard-risk (SR) and high risk (HR). High risk was defined as: residual tumor > 1,5 cm2, evidence of metastatic disease (stage M1-M4), and/or unfavorable histology (large cell/anaplastic) and/or MYC-MYCN amplification status [14]. The study was approved be the Swedish Ethical Review Authority (Dnr 2021 − 00803).

### Outcome

Acute toxicity was defined as side effects occurring during RT and within 2 months after RT (until start of adjuvant chemotherapy). Clinical side effects during RT were registered in RADTOX Quality Registry according to the RTOG (Radiation Therapy Oncology Group) classification. During the supplementary data collection from medical records classification of acute side effects were converted to CTCAE (Common Terminology Criteria for Adverse Events, version 5) to facilitate grading and comparability with earlier studies. Weight loss was graded according to CTCAE. Additional analysis on weight loss was conducted by comparing the percentual weight loss during treatment (nadir/baseline weight).

Hematological toxicity was defined as nadir during RT, graded according to CTCAE. To estimate the decrease in blood counts during RT, baseline blood counts prior to RT was compared to nadir during RT. Concurrent chemotherapy was regarded a potential confounding factor for bone marrow suppression although hematological toxicity of Vincristine is considered rare [9, 21, 22]. Subgroup analysis excluding all patients receiving concurrent chemotherapy was conducted to investigate the potential effect on both acute clinical symptoms and hematological toxicity.

### Radiotherapy

Photon CSI treatment was delivered with Three dimensional conformal radiation therapy (3D CRT) (*n* = 53), Tomotherapy (*n* = 11), Intensity modulated radiotherapy (IMRT) (*n* = 4) and Volumetric modulated arc therapy (VMAT) (*n* = 1). Proton therapy was delivered with Pencil beam scanning (PBS). Relative biological effectiveness (RBE) of protons was set to 1.1.

### Statistical methods

Descriptive statistics were used to analyze both categorical and continuous data. Categorical data were presented as percentages, while continuous data were summarized using medians with interquartile ranges. The chi-square test was used to determine whether there were significant differences in the distribution of clinical characteristics between the study groups for categorical variables, and Mann-Whitney test for continuous variables. To understand the potential differences between the groups regarding side effects logistic regression models were used with odds ratios (OR) and the corresponding 95% confidence intervals (CI). Progression-free survival (PFS) and overall survival (OS) was estimated using the Kaplan-Meier model with the corresponding log-rank test. PFS was defined as time from end of radiotherapy to first relapse (on MRI) or death, while OS was defined as time from end of radiotherapy to death due to any causes, and both outcomes were censored at last follow-up MRI. Statistical significance was considered with a P value of < 0.05. Data management and statistical analysis was carried out using R version 3.4.1.

## Results

### Patients and characteristics

Patients clinical and treatment characteristics are summarized in Table 1. Fifty-nine patients (61%) received photon-RT and 37 patients (39%) were treated with protons. Sixty-six patients were male (63%), and 39 patients (37%) were female. In the male population a higher number of patients received photon-RT compared to proton RT (30% vs. 70%, *p* = 0.02), In the female population the distribution was equal between RT techniques. No significant difference in age at diagnosis and performance status (ECOG) at start of treatment was demonstrated between groups.

Incidence of high-risk tumors, metastatic disease (M-stage) and histological subtype were comparable between the groups (Table 1). Prevalence of low-risk tumors (WNT-MB) was not possible to compare since WNT-analyze was available only for patients treated in recent years and often lacked in the photon group treated before 2015. Twenty-eight received chemotherapy prior to RT equally distributed between treatment groups. The most common treatment combination was Carboplatin, Vincristine, Etoposide and Methotrexate. Two patients (one from each treatment group) underwent autologous hematopoietic stem cell transplantation prior to radiotherapy. 54% of patients treated with photon-RT received concurrent chemotherapy compared to 14% of proton treated patients (p = < 0.001).

### Progression free and overall survival

**Fig. 1:**
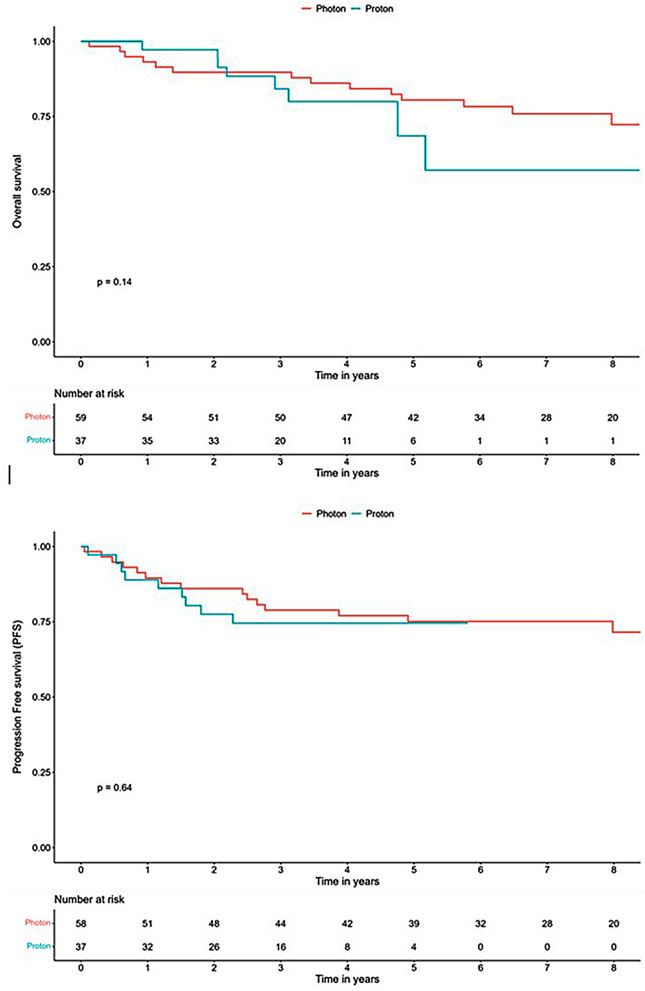
Overall survival (OS) and progression free survival (PFS) after proton and photon-RT

There was a significant difference in follow-up time between the photon and proton cohort (median 6.8 vs. 2.8 years; *p* < 0.001). There was no significant difference in PFS between the two cohorts, 5-year PFS photons 75,1% (95% CI 64,6–87,3) vs. protons 74,5% (95% CI 61,4–90,4). No significant difference in 5-year overall survival was observed between groups, photons 80,5% (95% CI 70.7–91.6) vs. protons 68.5% (95% CI 48.1–97.7) (Fig. 1). In total 27 cases of relapse occurred. Fourteen cases of relapses in the photon group (23.7%) and 9 (24.3%) in the proton group. Time to relapse was similar between groups (median 16.5 months (range 0.1–59.8) photon-RT vs. 14.1 months (range 1.3–27.8) proton-RT). No significant difference in patterns of failure was demonstrated between groups (Table 2).

### Dose distribution

Absorbed mean dose to intracranial OARs was significantly lower with proton-RT (Table 2). Proton-RT resulted in lower mean doses to the brainstem (*p* = 0.007) hippocampi and temporal lobes (*p* = < 0.001) compared to photon radiotherapy.

**Table 1:** Patient characteristics

	Photon (*n*=59)	Proton (*n*=37)	*p*-value
**Sex (%)**			
*Male*	44 (74.6)	19 (51.4)	0.020
*Female*	15 (25.4)	18 (48.6)	
**Age at diagnosis (year)**,** median [IQR]**	8.0 [6.0, 11.0]	7.0 [5.0, 10.0]	0.128
*range (year)*	2–17	2–15	
**ECOG Performance status before start of RT (%)**			
*0*	20 (33.9)	10 (27.0)	0.533
*1*	27 (45.8)	20 (54.1)	
*2*	7 (11.9)	6 (16.2)	
*3*	5 (8.5)	1 (2.7)	
**Hospital (%)**			
*Umeå University Hospital*	4 (6.8)	3 (8.1)	0.236
*Uppsala University Hospital*	9 (15.3)	4 (10.8)	
*Karolinska University Hospital*,* Stockholm*	12 (20.3)	13 (35.1)	
*Linköping University Hospital*	10 (16.9)	2 (5.4)	
*Göteborg University Hospital*	12 (20.3)	11 (29.7)	
*Skåne University Hospital*	12 (20.3)	4 (10.8)	
**Histology (%)**			
*Classic*	46 (78.0)	32 (86.5)	0.540
*Desmoplastic or nodular variant*	7 (11.9)	2 (5.4)	
*Anaplastic or large cell variant*	4 (6.8)	3 (8.1)	
*MD*	2 (3.4)	0 (0)	
**Risk (%)**			
*Standard*	40 (67.8)	22 (59.5)	0.406
*High risk*	19 (32.2)	15 (40.5)	
**M stage (%)**			
M0	43 (72.9)	29 (78.4)	0.728
M1	1 (1.7)	1 (2.7)	
M2	2 (3.4)	2 (5.4)	
M3	13 (22.0)	5 (13.5)	
**Chemotherapy before RT (%)**	14 (23.7)	14 (37.8)	0.139
**Concurrent Chemotherapy (%)**	32 (54.2)	5 (13.5)	< 0.001
*Vincristine*	31 (52.5)	4 (10.8)	
*Carboplatin*	1 (1.7)	1 (2.7)	
**Median CSI dose**,** Gy [IQR]**	23.4 [23.4, 35.2]	23.4 [23.4, 35.2]	0.737
*18 Gy (%)*	1 (1.7)	4 (10.8)	0.113
*23.4 Gy (%)*	40 (67.8)	20 (54.1)	
*≥35 Gy (%)*	18 (30.5)	12 (32.4)	
**Total dose to primary tumor (%)**			
54–55.8 Gy	50 (84.7)	36 (97.3)	0.050
>55.8 Gy	9 (15.3)	1 (2.7)	
**Surgery to RT interval**,** median [IQR]**	39.0 [29.0, 45.0]	34.5 [32.0, 42.0]	0.540
**Median follow-up time**,** years [IQR]**	6.84 [3.65, 9.25]	2.79 [1.80, 3.69]	<0.001
**Surgical radicality (%)**			
*Radical/GTR*	34 (57.6)	22 (59.5)	0.590
*<1.5 cm3*	6 (10.2)	7 (18.9)	
*≥1.5 cm3*	3 (5.1)	2 (5.4)	
*Metastatic*	15 (25.4)	6 (16.2)	
*MD*	1 (1.7)	0 (0.0)	
**Sedation during RT (%)**			
*No sedation*	19 (32.2)	12 (32.4)	0.266
*Anesthesia*	23 (39.0)	20 (54.1)	
*Sedation*	2 (3.4)	0 (0.0)	
*MD*	15 (25.4)	5 (13.5)	
**Blood levels at start of RT [IQR]**			
*Median baseline hemoglobine level (g/L)*	117.0 [112.0, 125.0]	108.0 [104.0, 115.0]	< 0.001
*Median baseline leukocytes level (x 109/L)*	5.8 [3.4, 7.0]	5.4 [3.9, 7.0]	0.870
*Median baseline platelet level (x 109/ µl)*	321.0 [246.0, 388.0]	280.0 [168.0, 357.0]	0.034
*Median baseline neutrophile level (x 109/ µl)*	2.7 [1.8, 3.9]	2.9 [1.8, 3.9]	0.572

Abbreviations: RT=Radiotherapy, ECOG= Eastern Cooperative Oncology Group, GTR=gross total resection, IQR=Interquartile range, RBE=Relative biologic effectiveness, MD=missing data

**Table 2:** Patient treatment characteristics

	Photon (*n*=59)	Proton (*n*=37)	*p*-value
**Doses to OARs**,** Gy**			
*Brainstem mean dose*,* median [IQR]*	52.90 [49.90, 53.90]	49.30 [46.90, 49.80]	0.007
*Hippocampi dx mean dose*,* median [IQR]*	46.92 [39.70, 50.34]	34.74 [30.10, 39.67]	<0.001
*Hippocampi sin mean dose*,* median [IQR]*	47.00 [39.70, 51.17]	33.69 [30.10, 41.73]	<0.001
*Temporal lobe dx mean dose*,* median [IQR]*	37.78 [34.22, 42.95]	26.74 [25.50, 28.56]	<0.001
*Temporal lobe sin mean dose*,* median [IQR]*	37.29 [34.04, 43.25]	26.22 [24.43, 29.45]	<0.001
**NG tube/PEG during RT (%)**			
*No need*	43 (72.9)	28 (75.7)	0.362
*NG tube/PEG*	3 (5.1)	4 (10.8)	
*IV Fluids*	2 (3.4)	0 (0.0)	
*Feeding tube prior to RT*	11 (18.6)	4 (10.8)	
**Cortison (%)**			
*No*	33 (55.9)	14 (37.8)	0.245
*Cortisone treatment initiated during RT*	24 (40.7)	21 (56.8)	
*Cortisone before start of RT*	2 (3.4)	1 (2.8)	
**Total number of relapse (%)**	14 (23.7)	9 (24.3)	0.633
*Isolated fossa posterior*	2 (14.3)	2 (22.2)	
*Isolated brain*,* other*	4 (28.6)	1 (11.1)	
*Isolated focal spine*	0 (0)	1 (11.1)	
*Diffuse or leptomeningeal disease*	6 (42.9)	4 (44.4)	
*Posterior fossa and focal spine*	2 (14.3)	1 (11.1)	

Abbreviations: OARs= Organs at risk, Gy= Gray, IQR=Interquartile range, RT= Radiotherapy, NG=Nasogastric, PEG=Percutaneous Endoscopic Gastrostomy, IV=Intravenous

### Acute clinical symptoms

No significant difference was demonstrated in incidence or severity of fatigue, headache, nausea, dermatitis or gastrointestinal toxicity during treatment. Incidence of grade 2 nausea (24.3% vs. 25.4%) and grade 3 nausea (18.9% vs. 16.9%) was similar in the proton and photon group (*p* = 0.71). Incidence of grade 1 headache (18.9% vs. 23.7%) and grade 2 headache (2.7% vs. 3.4%) was also comparable between groups (*p* = 0.6) (Table 3).

No significant difference in weight loss was demonstrated between groups graded according to CTCEA (Table 3). Median percentual weight loss during treatment was − 4.66% (range (5.2%- (-15.1%) in the photon cohort and − 1.9% (range (8.0%-(-12.6%) in the proton group resulting in a borderline significant difference in percentual weight loss between treatment groups (*p* = 0.09).

Multivariate regression analysis regarding CSI dose and concurrent chemotherapy as possible confounding factors demonstrated no difference in incidence or severity of acute symptoms between groups. The use of sedation and anesthesia during radiotherapy was analyzed since sedation during RT could contribute to nausea but no difference was observed between groups (Table 1). The brainstem dose was analyzed to investigate potential correlation with development of nausea. A significant difference in brainstem dose was observed between treatment modalities (photons 52.9 Gy vs. protons 49.3 Gy, *p* = 0.007), however the result did not correlate with lower incidence of nausea in the proton group.

### Supportive treatment during RT

During RT 45 patients (46.9%) received cortisone for treatment related side effects. No significant difference in cortisone use was observed between the proton and photon group, 56.8% vs. 40.7% (*p* = 0.25). No significant difference was observed between treatment cohorts regarding nutritional support with IV fluids or feeding tube (*p* = 0.36) (Table 2).

**Table 3:** Acute toxicity

Variable	CTCEA	Photon (*n*=59)	Proton (*n*=37)	*p*-value
Fatigue (%)	0	33 (55.9)	19 (51.4)	0.539
	1	20 (33.9)	16 (43.2)	
	2	6 (10.2)	2 (5.4)	
Headache (%)	0	43 (72.9)	28 (75.7)	0.595
	1	14 (23.7)	7 (18.9)	
	2	2 (3.4)	1 (2.7)	
	3	0 (0.0)	0 (0.0)	
	MD	0 (0.0)	1 (2.7)	
Nausea (%)	0	9 (15.3)	7 (18.9)	0.710
	1	25 (42.4)	13 (35.1)	
	2	15 (25.4)	9 (24.3)	
	3	10 (16.9)	7 (18.9)	
	MD	0 (0.0)	1 (2.7)	
Dermatitis (%)	0	21 (35.6)	17 (45.9)	0.279
	1	26 (44.1)	18 (48.6)	
	2	9 (15.3)	2 (5.4)	
	MD	3 (5.0)	0 (0.0)	
Lower bowel (%)	0	54 (91.5)	36 (97.3)	0.256
	1	5 (8.5)	1 (2.7)	
	2	0 (0.0)	0 (0.0)	
Weigth loss (%)	0 (<5%)	23 (39.0)	22 (59.5)	0.226
	1 (5 -<10%)	19 (32.2)	8 (21.6)	
	2 (10 - <20%)	5 (8.5)	4 (10.8)	
	3 (>=20%)	0 (0.0)	0 (0.0)	
	MD	12(20.3)	3 (8.1)	
**Hematologic toxicity**				
Leukopenia (%)	0	4 (6.8)	6 (16.2)	0.612
	1	4 (6.8)	3 (8.1)	
	2	20 (33.9)	15 (40.5)	
	3	19 (32.2)	11 (29.7)	
	4	4 (6.8)	1 (2.7)	
	MD	8 (13.6)	1 (2.7)	
Neutropenia (%)	0	10 (16.9)	9 (24.3)	0.649
	1	7 (11.9)	5 (13.5)	
	2	18 (30.5)	13 (35.1)	
	3	10 (16.9)	8 (21.6)	
	4	6 (10.2)	1 (2.7)	
	MD	8 (13.6)	1 (2.7)	
Anemia (%)	0	15 (25.4)	17 (45.9)	0.219
	1	20 (33.9)	8 (21.6)	
	2	15 (25.4)	11 (29.7)	
	3	1 (1.7)	0 (0.0)	
	MD	8 (13.6)	1 (2.7)	
Thrombocytopenia (%)	0	36 (61.0)	30 (81.1)	0.085
	1	10 (16.9)	1 (2.7)	
	2	4 (6.8)	5 (13.5)	
	3	1 (1.7)	0 (0.0)	
	MD	8 (13.6)	1 (2.7)	

Abbreviations: MD=Missing data

**Fig. 2:**
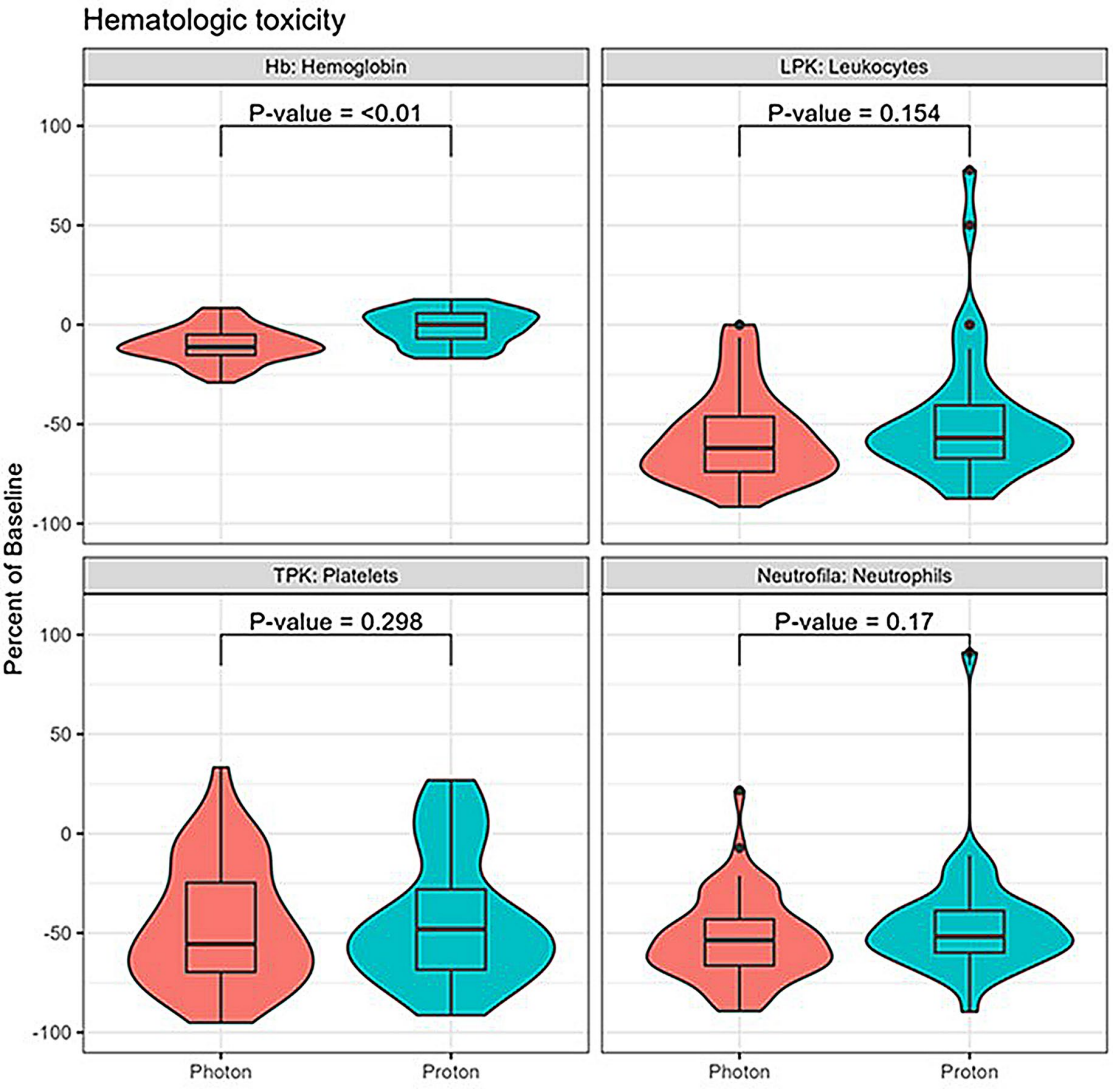
Decrease in blood levels during radiotherapy. Median percentage of decrease in hemoglobin, leucocytes, platelets and neutrophils comparing baseline levels and nadir during treatment. A significant difference in decrease of hemoglobin was demonstrated between photon and proton treatment. No difference in reduction of LPK, TPK or Neutrophils during photon and proton radiotherapy

### Hematological toxicity

Graded according to CTCEA no significant difference was demonstrated in bone marrow suppression between treatment groups (Table 3).

In subgroup analysis excluding all patients with concurrent chemotherapy no difference was observed in incidence of grade ≥ 2 toxicity between protons and photons; anemia 21.9%( n 7) vs. 11.5% (n 3) (*p* = 0.492), leukopenia 71.9% (n 23) vs. 76.9% (n 20) (*p* = 0.892), thrombocytopenia 12.5% (n 4) vs. 15.4% (n 4) (*p* = 0.869) and neutropenia 59.4% (n 19) vs. 57.7% (n 15) (*p* = 1.0).

In analyze of percentual decrease in hemoglobin (nadir/baseline) a significant difference was observed between photon-RT and proton-RT. Median percentual decrease in red blood counts during photon-RT was − 11.25% (range 8.5%-(-29.1%) compared to 0% (range 12.4%-(-17.0%) in the proton-RT group. (p = < 0.001) In subgroup analyze excluding patients with concurrent chemotherapy the significant difference in hemoglobin decrease remained (*p* = 0.0067). No difference was observed between treatment groups in percentual decrease of leukocytes, platelets of neutrophiles (Fig. 2).

## Discussion

In our study no significant difference was observed between photon and proton-RT in incidence or severity of acute symptoms. Multivariate regression analyze regarding CSI dose as confounding factor was conducted and demonstrated the same results. The use of concurrent chemotherapy in the photon group was significantly higher than in the proton group, 56% vs. 14% (p = < 0.001). To address concurrent chemotherapy as a possible confounding factor for acute toxicity such as nausea, weight loss, fatigue and hematologic toxicity, a subgroup analyze was made excluding all patients receiving concurrent chemotherapy. No difference in acute toxicity was observed in the subgroup analyze regarding acute symptoms. A borderline difference (*p* = 0.09) was observed in median percentual weight loss during RT, (-4.7% photon-RT vs. -1.9% proton-RT) but no higher incidence of nausea, need for supportive treatment with cortisone or nutritive intervention was observed that would support a higher incidence of weight loss in the photon group.

A few studies have previously analyzed acute side effects during photon and proton-CSI, but since medulloblastoma is a rare disease studies available are often made on small cohorts and often includes different types of brain tumors. A study by Uemura et al. 2022 evaluating acute toxicity after CSI radiotherapy for 62 patients ≤ 18 years with brain tumors demonstrated a lower incidence of > grade 2 nausea during proton CSI-RT (n 26) compared to photon CSI-RT (n 36) [10]. In a similar study by Brown et al. 2013 comparing acute toxicity during CSI-RT in 40 adult medulloblastoma patients, the proton CSI treatment group experienced less grade 2 nausea compared to patients treated with photon-CSI. Weight loss during RT was also lower in the proton group (1.2% vs. 5.8%) and weight loss > 5% during treatment was less common among patients receiving protons [18]. A study by Song et al. (2014) analyzing acute toxicity of CSI-RT in 43 patients < 18 years with brain tumors the incidence of diarrhea was higher in the photon CSI group, 23% (n 3) compared to no patients with diarrhea in the proton CSI group (*p* = 0.023) [23].

The different outcome in acute toxicity compared to previous studies might be explained by differences in the studied cohorts. In the study by Uemura et al. concurrent chemotherapy was common, 92.3% of proton-CSI patients and 77.8% of photon-CSI patients. Concurrent chemotherapy consisted of different regimes such as cisplatin/cyclophosphamide, weekly Vincristine, Temozolomide, Etoposide, Irinotecan and Topotecan. The study included different types of brain tumors such as ETMR (Embryonal tumor with multilayered rosettes), germ cell tumor, AT/RT (Atypical Teratoid/Rhabdoid Tumor) with both supratentorial and infratentorial primary sites [10]. The use of different concurrent chemotherapy and tumor location might have affected the incidence and severity of acute toxicity during RT. In the study by Brown et al. Vincristine was used as concurrent chemotherapy (photon-RT 26% (n 5) and proton-RT 24% (n 5)). Regarding concurrent chemotherapy, the studied cohort was more comparable to our cohort. On the other hand, an adult population was investigated by Brown et al. and the applicability of the results on a paediatric population is uncertain.

Hematological toxicity is a common adverse event of CSI irradiation as the targeted structure covers a large volume of the vertebra. Proton technique offers sparing of hematopoietic bone marrow and previous studies have shown less hematological toxicity during CSI irradiation with proton therapy. In a study by Song et al. 2014 craniospinal irradiation with proton therapy was associated with less severe thrombocytopenia (less grade 3–4 toxicity) compared to photon-RT [23]. According to a study by Liu et al. 2020 comparing proton and photon-CSI irradiation lymphocyte counts remained higher during proton radiotherapy treatment compared to photon therapy. Photon treatment was associated with higher incidence of grade ≥ 3 leukopenia, grade ≥ 2 anemia and grade ≥ 1 thrombocytopenia [9]. In the study by Brown et al. comparing proton and photon CSI treatment in an adult population, proton-CSI was associated with smaller reduction in white blood cells, hemoglobin and platelets [18]. A study by Yoo et al. (2021) analyzing acute hematological outcome after proton and photon-CSI in 66 paediatric patients with brain tumors demonstrated no significant difference in hemoglobin decline between treatment groups but a significantly lower rate of grade 3 anemia in the proton-CSI group. The study also demonstrated lower decline and better recovery of total lymphocytes and platelets with proton-CSI [24].

In our study no difference in hematological toxicity was observed between groups when categorizing toxicity as nadir during treatment according to CTCAE. This method of analyzing hematological toxicity does not take baseline values into consideration. To investigate the actual reduction of blood counts during RT the percentual reduction during treatment was analyzed (nadir/baseline). The percentual reduction of hemoglobin was significantly lower in the proton-RT group (p = < 0.001) (Fig. 2). The same result was demonstrated after excluding patients receiving concurrent chemotherapy (*p* = 0.0067). This indicates a benefit of proton-CSI maintaining hemoglobin levels during radiotherapy compared to photon-CSI. Since proton-CSI enables vertebral body-sparing technique with partial radiation of the vertebra one could have expected a greater difference in hematological toxicity between treatment groups. However, in growing children, vertebral body-sparing technique can potentially increase the incidence of spinal deformity and therefore a whole vertebral body irradiation is often preformed regardless of the existing technique. In the study by Brown at el an adult population was investigated and all patients received vertebral body-sparing technique. The study presented smaller reduction in white blood cells, hemoglobin and platelets with protons compared to photon-RT, which demonstrates the potential benefit of proton-CSI. In our study only 30% of the proton treated patients (n 11) received vertebral body-sparing CSI and we believe this might have impacted the low difference in hematological toxicity between treatment groups.

Regarding 5-year OS and PSF, data in this study is still immature. Follow-up time for proton-RT patients was significantly shorter than for photon-RT patients (median 2.8 vs. 6.8 years; *p* < 0.001) and longer follow-up time is required for the proton treated group to analyze and draw conclusions regarding survival data. Five-year OS in the photon group was 80.5% vs. 68.5% in the proton group but the difference was affected by the low number of proton patients with 5-year follow-up time. Five-year PFS was similar between groups (photon 75,1% vs. protons 74,5%) which indicates comparable outcome in PFS between treatment groups (Fig. 1). However, no significant statistical difference in OS or PFS was demonstrated. Patterns of failure was similar between photon-RT and proton-RT. In total 23 (24.0%) cases of recurrent disease occurred, the most common site of failure was diffuse or leptomeningeal disease (Table 2). Isolated posterior fossa failure was relatively rare, 17.4% (*n* = 4). Previous studies have reported similar results with disseminated failure rate of 64–68% and local fossa posterior failure rate of 9–15% [15, 25]. Longer follow-up data is needed to compare outcome in patterns of failure between the treatment groups.

This study is a national multicenter study with almost 100% coverage of patients treated for medulloblastoma during 2008–2020 in Sweden. To our knowledge this study is the largest analysis so far on acute side effects of proton and photon-RT for paediatric medulloblastoma. A strength with the study was that only patients with medulloblastoma were included, resulting in a homogeneous cohort. However, the present study has several limitations. There was a higher number of patients receiving photon-RT and follow-up time after RT was significantly longer in the photon group. Data was retrospectively collected, although all data was analyzed and classified by the same investigator contributing to similar grading of symptoms. Concurrent chemotherapy was more common in the photon RT group which might have impacted symptoms, sub analysis was made to evaluate concurrent chemotherapy as a confounding factor. Antiemetics were often prescribed to patients, to use when necessary during radiotherapy. Because of the difficulty in a retrospective study to assess whether patients used the prescribed antiemetics or not, cortisone use was regarded a more reliable variable to compare the need for intervention due to nausea, and therefore analyzed in this paper. Patients suffering from nausea were first treated with antiemetics, most commonly Ondansetron, and in case of more severe nausea, cortisone (Betamethasone). Supportive treatment with antiemetics, cortisone and nutritive support depended on the physician´s choice and might have affected expression of symptoms such as nausea, weight loss and headache. All proton patients received treatment at the same treatment center (Skandion Clinic, Uppsala). Local traditions in cortisone use and nutritive support might have impacted the expression of acute symptoms during treatment. In this study we lack information of blood transfusion during treatment. Differences in transfusion limits and traditions at different treatment centers could potentially have impacted the outcome.

We demonstrate that proton radiotherapy is safe and well tolerated treatment. However, the most benefit from proton radiotherapy is expected years after treatment on development of late complications. A study by Kahalley et al. (2019) demonstrated superior long-term outcome in global intelligence quotient (IQ), perceptional reasoning and working memory in paediatric patients with medulloblastoma treated with proton therapy compared with photon radiotherapy [12]. In this study we demonstrate a superior dose distribution with proton-RT with significantly lower radiation doses to cranial OAR´s, which hopefully also will translate into lower risk of long-term cognitive impairment. With reduced radiation dose to healthy tissue, proton-RT could potentially reduce risk of long-term complications. To establish proton radiotherapy as state of the art treatment for medulloblastoma, further investigation of long-term side effects, including evaluation of cognitive impairment, secondary malignancies, hormonal deficiency and survival data is warranted.

## Data Availability

Data will be made available from the corresponding author on reasonable request.
