# Correction to: Acute side effects of proton and photon radiotherapy for medulloblastoma: a retrospective national multicenter study

**DOI:** 10.1007/s11060-025-05235-2

**Published:** 2025-09-29

**Authors:** Linn Söderlund Diaz, Måns Agrup, Anna Asklid, Anna Embring, Jacob Engellau, Ingrid Fagerström Kristensen, Anna Flejmer, Charlotta Fröjd, Martin P Nilsson, Anna Maja Svärd, Angelica Walfridsson, Malin Blomstrand

**Affiliations:** 1 Department of Oncology, Sahlgrenska University Hospital, Blå Stråket 2, Gothenburg, 413 46 Sweden; 2 Department of Oncology, Linköping University Hospital, Linköping, Sweden; 3 Department of Oncology, Karolinska University Hospital, Stockholm, Sweden; 4 Department of Oncology-Pathology, Karolinska Institute, Stockholm, Sweden; 5 Department of Radiation Oncology, Skåne University Hospital, Lund, Sweden; 6 Department of Radiation Oncology, Uppsala University Hospital, Uppsala, Sweden; 7 Department of Oncology, Umeå University Hospital, Umeå, Sweden; 8 Sahlgrenska Academy, Institute of Clincial Sciences, Department of Oncology, Gothenburg, Sweden

**Correction: Journal of Neuro-Oncology (2025) 173:559–569**

10.1007/s11060-025-05016-x

In this article the third author’s name Asklid was incorrectly written as Askild.

The original article has been corrected.

